# Protocol registration issues of systematic review and meta-analysis studies: a survey of global researchers

**DOI:** 10.1186/s12874-020-01094-9

**Published:** 2020-08-25

**Authors:** Gehad Mohamed Tawfik, Hoang Thi Nam Giang, Sherief Ghozy, Ahmed M. Altibi, Hend Kandil, Huu-Hoai Le, Peter Samuel Eid, Ibrahim Radwan, Omar Mohamed Makram, Tong Thi Thu Hien, Mahmoud Sherif, As-Saba Hossain, Tai Luu Lam Thang, Livia Puljak, Hosni Salem, Tarek Numair, Kazuhiko Moji, Nguyen Tien Huy

**Affiliations:** 1 Faculty of Medicine, Ain Shams University, Cairo, Egypt; 2 http://www.onlineresearchclub.org/; 3 The University of Da Nang, Da Nang, Vietnam; 4 Neurosurgery Department, El Sheikh Zayed Specialized Hospital, Giza, Egypt; 5 Henry Ford Allegiance Health, Henry Ford Health System, Jackson, MI USA; 6 Faculty of Medicine, Menofia University, Menofia, Egypt; 7 Saigon General Hospital, Ho Chi Minh City, Vietnam; 8 Faculty of Medicine, October 6 University, Giza, 12566 Egypt; 9 School of Medicine, Viet Nam National University, Ho Chi Minh City, Vietnam; 10 Faculty of Medicine, Al-Azhar University, Cairo, Egypt; 11 Dhaka Medical College, Dhaka, Bangladesh; 12 Faculty of Medicine, Pham Ngoc Thach University, Ho Chi Minh City, Vietnam; 13 Department of Anatomy, Histology and Embryology, University of Split School of Medicine, Soltanska 2, 21000 Split, Croatia; 14 Urology Department, Faculty of Medicine, Cairo University, Giza, Egypt; 15 School of Tropical Medicine and Global Health, Nagasaki University, Nagasaki, 852-8523 Japan; 16 Institute of Research and Development, Duy Tan University, Da Nang, 550000 Vietnam

**Keywords:** Registration, PROSPERO, Idea theft, Duplication, Systematic review, Meta-analysis

## Abstract

**Background:**

Although protocol registration of systematic reviews/meta-analysis (SR/MA) is still not mandatory, it is highly recommended that authors publish their SR/MA protocols prior to submitting their manuscripts for publication as recommended by the Cochrane guidelines for conducting SR/MAs. our aim was to assess the awareness, obstacles, and opinions of SR/MA authors about the protocol registration process.

**Methods:**

A cross-sectional survey study included the authors who published SR/MAs during the period from 2010 to 2016, and they were contacted for participation in our survey study. They were identified through the literature search of SR/MAs in Scopus database. An online questionnaire was sent to each participant via e-mail after receiving their approval to join the study. We have sent 6650 emails and received 275 responses.

**Results:**

A total of 270 authors responses were complete and included in the final analysis. Our results has shown that PROSPERO was the most common database used for protocol registration (71.3%). The registration-to-acceptance time interval in PROSPERO was less than 1 month (99.1%). Almost half of the authors (44.2%) did not register their protocols prior to publishing their SR/MAs and according to their opinion that the other authors lack knowledge of protocol importance and mandance to be registered, was the most commonly reported reason (44.9%). A significant percenatge of respondents (37.4%) believed that people would steal their ideas from protocol databases, while only 5.3% reported that their SR/MA had been stolen. However, the majority (72.9%) of participants have agreed that protocol registries play a role in preventing unnecessary duplication of reviews. Finally, 37.4% of participants agree that SR/MA protocol registration should be mandatory.

**Conclusion:**

About half of the participants believes that the main reason for not registering protocols, is that the other authors lack knowledge concerning obligation and importance to register the SR/MA protocols in advance. Therefore, tools should be available to mandate protocol registration of any SRs beforehand and increasing awareness about the benefits of protocol registration among researchers.

## Background

Systematic reviews and meta-analyses (SR/MAs) are a form of literature reviews that aim to answer a particular research question by collecting and critically analyzing all empirical evidence published as primary research studies [1, 2]. Systematic reviews and meta-analyses (SR/MAs) are deemed to be on the top of the evidence hierarchy in medicine [1]. Recently, it is obvious that the number of published SR/MAs is soraing as compared to primary studies [3]. This trend could be justified as SR/MAs may be easier to perform, require lower funding, and may be less time-consuming compared to primary research [4].

Along with this exponential growth, “duplicate” or “overlapping” work is absolutely concerning, as the same research question is being addressed and the results get published by different authors, in a short period of time [5–7]. Interestingly, these reviews do not necessarily reach the same conclusions or including the same studies [8]. Although replication of prior research does not need to be negative, unnecessary and redundant duplication should direct researchers to carefully review existing evidence, and ascertain the added value of any new work as an initial step [9, 10].

On the other hand, overlapping studies are sometimes produced as researchers may not be aware of other ongoing, but still unpublished SR/MAs. Hence, efforts are made to encouarge the SR/MA study protocols registration in publicly-available databases to allow researchers to identify existing protocols of ongoing reviews [11, 12]. Several databases offer the researchers a place to register their protocols in, e.g. PROSPERO University of York, Cochrane Database of Systematic Reviews, Campbell Collaboration, yet the most known between researchers is PROSPERO. In February 2011, the Centre for Reviews and Dissemination (CRD) at the University of York founded PROSPERO – an international database for registering SR/MA protocols – which aims to promote high methodological standards, ensure transparency of review process, and reduce undesirable duplication [13]. PROSPERO has received wide acceptance among the research community, resulting in its continuous expansion [13]. PROSPERO registrations are increasing rapidly especially in the period 2011–2017 and expected this expansion will reach over 30,000 registrations by the end of 2017 [14].

Although registering the protocols of SR/MAs is not mandated by medical journal editors, unlike the situation with clinical trial, registration of SR protocols is now recommended by the National Institute for Health Research (NIHR) [12, 15–17]. However, according to a recent study, the registration rate of SRs’ protocols is still low (only 21%) [17]. Meanwhile, an investigation conducted in 2019 revealed that the rate of registering the protocols of “dose-response” meta-analyses (DRMAs) was even lower, reaching about 8.51% from a total of 529 investigated DRMAs [18]. This may partially be attributed to the fact that PROSPERO is an open-access register, which may raise the concern about the possibility of idea theft where somebody else reproduces original ideas from the published protocols [13]. Therefore, our aim is to investigate the knowledge and attitude of systematic reviews-publishing authors about the process of registering their protocols in PROSPERO. We also asked recruited participants about their perception of current solutions to issues related to the usage of PROSPERO as well as their recommendations for better protocol registries. To the best of our knowledge, the literature lacks studies in this regard so this will be the first study to address this gap in the literature.

## Methods

### Participants

Participants in this study were corresponding authors who had published SR/MA. Potential participants were identified thru two steps. In the first step, we conducted a search for SR/MAs published in Scopus between 2010 and 2016. This time frame was chosen because of sharply increased in the number of systematic review in this period, therefore, we selected this period to collect information about SR/MA authors from it [19]. We followed the reporting online surveys Checklist for Reporting Results of Internet E-Surveys (CHERRIES) guidelines which is found in Supplementary Table 1 [20]. We searched for the related reviews using the following search terms: ((“systematic review” OR “systematic literature review” OR “meta-analysis”) OR (Cochrane Database Syst Rev)) in Scopus database. We have not applied ny restrictions on the topic, country, or language of SR/MAs. Scopus restricts the number of references that can be obtained to a maximum of 2000 in each year and our aim was to target at least 10,000 authors. So, we collected 14,000 SR/MA manuscripts from seven-year periods (2010–2016) according to our criteria. To make sure that we will reach that target number, we searched for SR/MAs published in the seven-year period. As a second step, we screened retrieved articles for potential SR/MAs in abstract and title, then full-text screening steps to exclude any non SR/MA manuscripts from them. Then we collected 10,469 SR/MA manuscripts for different author emails and extracted data about their corresponding authors from included SR/MAs. From each of the 10,469 SR/MAs, we extracted authors’ information such as names, study title, year of publication, author affiliations, and e-mail of corresponding authors using Endnote software to export these details from selected results in Scopus and exclude duplications.

### Data collection

**Fig. 1:**
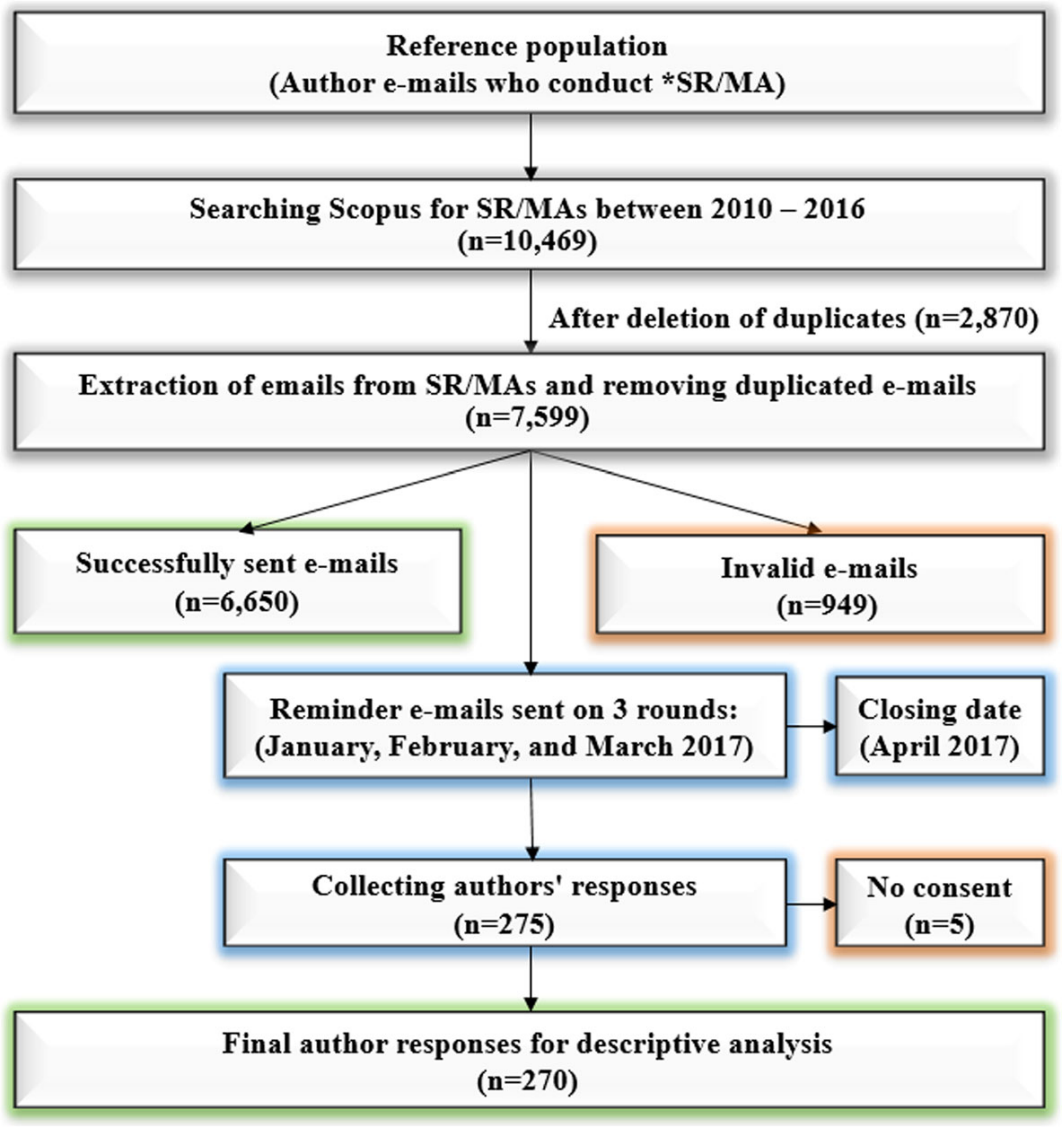
Flow chart of participants’ selection process

An online questionnaire was sent to the SR/MAs authors via e-mail in three rounds starting from January 2017. After the initial e-mail, we sent two more reminders with a one-month period between each e-mail. We finished receiving e-mails from the participants in April 2017. In our study, we performed no clinical intervention on human participants. A local ethics committee in Nagasaki University ruled that no formal ethics approval was required in this particular case, as we cannot identify each participant in our online survey (Supplementary Fig. 1). A consent was obtained from the participants at the beginning of the study. We stated at the last sentence of the sent online cover letter of our questionnaire: “By completing and submitting this survey, you are indicating your consent to participate in the study. Your participation is appreciated.” Responses were only included in our analysis if participants gave a ‘yes’ response to the following question ‘Have you read information about the study given in the cover letter and agree to participate in this survey?’. Failure to obtain a consent has resulted in exclusion this participant. The number of potential participants, to whom our e-mails could not be delivered, was recorded. A flow chart has been made to depict the process of participants recruitment (Fig. 1).

### Development of the online questionnaire

Our survey consisted of two sections. The first section involved some questions about the participants’ demographic characteristics. The second section comprised a comprehensive questionnaire designed to gather information about authors’ knowledge, opinions, practice, and fear of idea theft related to protocol registration.

The questionnaire was composed of 38 items divided into the following sections: 1) demographic and professional information about authors (items 1–8): age, gender, author’s work country, years of experience in the SR/MA field, fields of interest, number of published SR/MA, the highest impact factor and their roles in research studies, 2) SR/MA registration (items 9–19): databases for SR/MA protocol registration, the proportion of registered SR/MA, proportion of registered SR/MA protocols that was not published, opinions about the reason for not registering SR/MA, reason for not publishing registered SR/MA and information about protocol registration process, 3) opinions regarding registration protocol and problem of duplication (items 20–36). Finally, we added an open-ended question to gather other opinions regarding the protocol registration process, which were not covered by our previous questions (Supplementary file 1). We have pretested the questionnaire to assess its quality by 37 researchers in our group, who had authored and published a SR/MA.

### Data analysis

Descriptive analysis was carried out using R software version 3.4.1. R Core Team (2013). R: A language and environment for statistical computing. R Foundation for Statistical Computing, Vienna, Austria. ISBN 3–900,051–07-0, URL: http://www.R-project.org/. The packages used are “psych” and “models”.

## Results

We downloaded 10,469 articles from Scopus and retrieved the e-mails from them technically (not manually). After removing duplicated emails, there were 7599 e-mails available. The initial email sent to 7599 authors, but were delivered successfully to 6650 authors after invalid 949 emails (Fig. 1). We received a response to the survey from 275 authors from 6650 successfully sent emails with response rate (4.14%). However, we excluded from the analysis five authors who responded in the survey that they are not giving a consent to participate, Finally, we included 270 SR/MA responses in our analysis.

### Baseline and demographic characteristics

Most of the authors were men, with a mean age of 45.2 years. The highest number of authors came from four countries: 47 authors from USA (18%), 32 from UK (12.2%), 23 from Italy (8.7%), 16 from the Netherlands and Canada equally (6.1%) (Table 1). Most of the respondents had classified clinical trials on the top of their research intrests, followed by epidemiology, and diagnostic accuracy. On average, they had nine years of experience in SR/MAs. Most of the authors (190) had published range 1–10 papers (72.2%), while 63 authors had published range 10–50 papers (24%). When assessing the highest impact factor (IF) for journals in which the authors managed to publish, the majority managed to publish in journals with IF ranging between 2 and 5, followed by those with IF ranging between 5 and 10.

**Table 1:** Demographic and professional characteristic of participants

	*Number (%)*
Age, mean (SD), *n* = 249	45.2 (11.6)
Men, *n* = 266	172 (64.7)
Country, *n* = 262	
➣ USA	47 (18)
➣ UK	32 (12.2)
➣ Italy	23 (8.7)
➣ Netherlands	16 (6.1)
➣ Canada	16 (6.1)
➣ Australia	15 (5.7)
➣ Brazil	12 (4.6)
➣ Others –each country less than all mentioned above –	101 (38.6)
Continent, n = 262	
➣ WHO Eastern Mediterranean Region	12 (4.6)
➣ WHO European Region	136 (52)
➣ WHO African Region	2 (0.8)
➣ WHO Region of the Americas	75 (28.6)
➣ WHO South-East Asia Region	8 (3.1)
➣ WHO Western Pacific Region	29 (11.1)
Years of experience in SR/MA, mean (SD), *n* = 265	9.2 (6.3)
Main research interest of SR/MA, *n* = 264	
➣ Clinical trial	130 (49.2)
➣ Epidemiology	43 (16.2)
➣ Diagnostic accuracy	18 (6.8)
➣ Basic science (such as in vitro study)	14 (5.3)
➣ Genetic association	9 (3.4)
➣ Other	50 (18.9)
Number of publications in SR/MA, mean (SD), *n* = 263	15.1 (52.2)
	**N (%)**
➣ 1–10	190 (72.2)
➣ 10–50	63 (24)
➣ 50–100	7 (2.7)
➣ > 100	3 (1.1)
Highest Impact Factor Journal of your publications in SR/MA, *n* = 267	
➣ 0–2	36 (13.6)
➣ 2–5	84 (31.5)
➣ 5–10	76 (28.5)
➣ 10–20	30 (11.4)
➣ > 20	15 (5.6)
➣ Do not know	26 (9.7)

*SD: standard deviation, SR/MA: systematic review/meta-analysis*

### Protocol registration process

Upon assessing how often protocols were being registered, 44.2% of participants never registered their protocols before starting their SR/MA while only 10.1% reported that they registered all of their protocols. The most commonly reported reasons for not registering protocols were “not knowing that protocols should be registered” (44.9%), followed by “registration is not mandatory” (43%), “not knowing the benefits of registration” (35%), “registration is a time-consuming process” (32.7%), and fear that somebody will steal their ideas (24.3%). The majority of participants (77%) said that SR/MA protocol registration was not required at their institutes.

Meanwhile, PROSPERO and Cochrane Database of Systematic Reviews were the most commonly used databases for publication of the SR/MA protocols (71.3 and 45.3%, respectively). Regarding the registration-to-acceptance interval, 78.6% of authors who applied for registration indicated that their protocols were accepted by PROSPERO within one week of registration and 99.1% of protocols were accepted within one month. Only 1.6% of authors experienced rejection by PROSPERO upon submitting their SR/MA protocols.

Out of those who had registered their protocols, about 80% eventually managed to publish their manuscripts. The mean registration-to-submission interval was 11.1 ± 8.1 months. The two primary reasons for not publishing the registered SR/MA were (i) not completing the ongoing projects and (ii) not reaching a favorable conclusion (Table 2).

**Table 2:** The occurrence of protocol registration

	*Number (%)*
Site of previous SR/MA protocols register, *n* = 150 (checklist choice)	
➣ PROSPERO University of York	107 (71.3)
➣ Cochrane Database of SRs (CDSR)	68 (45.3)
➣ Campbell Collaboration	4 (2.7)
➣ Other	13 (8.7)
Proportion of SR/MA that was registered before starting, *n* = 267	
➣ 100%	27 (10.1)
➣ 80–100%	21 (7.9)
➣ 50–80%	32 (12)
➣ 20–50%	27 (10.1)
➣ < 20%	42 (15.7)
➣ None of them	118 (44.2)
Proportion of SR/MA that was registered, but never come to publish, *n* = 260	
➣ 100%	2 (0.8)
➣ 80–100%	1 (04)
➣ 50–80%	1 (0.4)
➣ 20–50%	10 (3.8)
➣ < 20%	38 (14.7)
➣ None of them	190 (80)
Reason for not publishing registered SR/MA, *n* = 185 (checklist choice)	
➣ Have not reached the favorable conclusion	48 (26)
➣ Have not finished	129 (69.7)
➣ Pressure from sponsor/contractor	18 (9.7)
➣ Other	46 (24.9)
Ever conducted SR/MA of basic biomedicine, such as in vivo or in vitro studies? *n* = 267	43 (16.1)
If yes, did you register the protocol, *n* = 96	
➣ Yes	9 (9.4)
Have you been rejected by PROSPERO when submitted a SR/MA protocol in basic biomedicine?, *n* = 191	3 (1.6)
If yes, What did you do when you got the rejection, *n* = 18	
➣ Contact PROSPERO University of York	2 (11.1)
➣ Revise and re-submit it to PROSPERO without any contact	0 (0)
➣ Other	16 (88.9)
Duration for the PROSPERO to accept protocol?, *n* = 107	
➣ 1 to 3 working days	62 (58)
➣ 4 to 6 working days	22 (20.6)
➣ 1 week to 1 month	22 (20.6)
➣ > 1 month	1 (1)
Average duration (in months) from registration to submission of your SR/MA manuscript, mean (SD), *n* = 115	11.1 (8.1)
Is registration of SR/MA protocol required in your institution?, *n* = 247	
➣ Yes	15 (6.1)
➣ No	190 (77)
➣ Do not know	42 (17)
Are your SR/MA protocols agreed by sponsors?, *n* = 206	
➣ Yes	53 (26)
➣ No	53 (26)
➣ I do not inform the sponsor	100 (48)
Opinion on reason for not registering SR/MA, n = 263 (checklist choice)	
➣ Submitting protocol takes too much time	86 (32.7)
➣ Afraid of others stealing ideas	64 (24.3)
➣ Did not know that it should be registered	118 (44.9)
➣ Have no idea about the benefit of registering the protocol	92 (35)
➣ It is not mandatory	113 (43)
➣ Other	30 (11.41)

*SR/MA: systematic review meta-analysis*

### Authors’ opinions on registration protocol of SR/MA

**Fig. 2:**
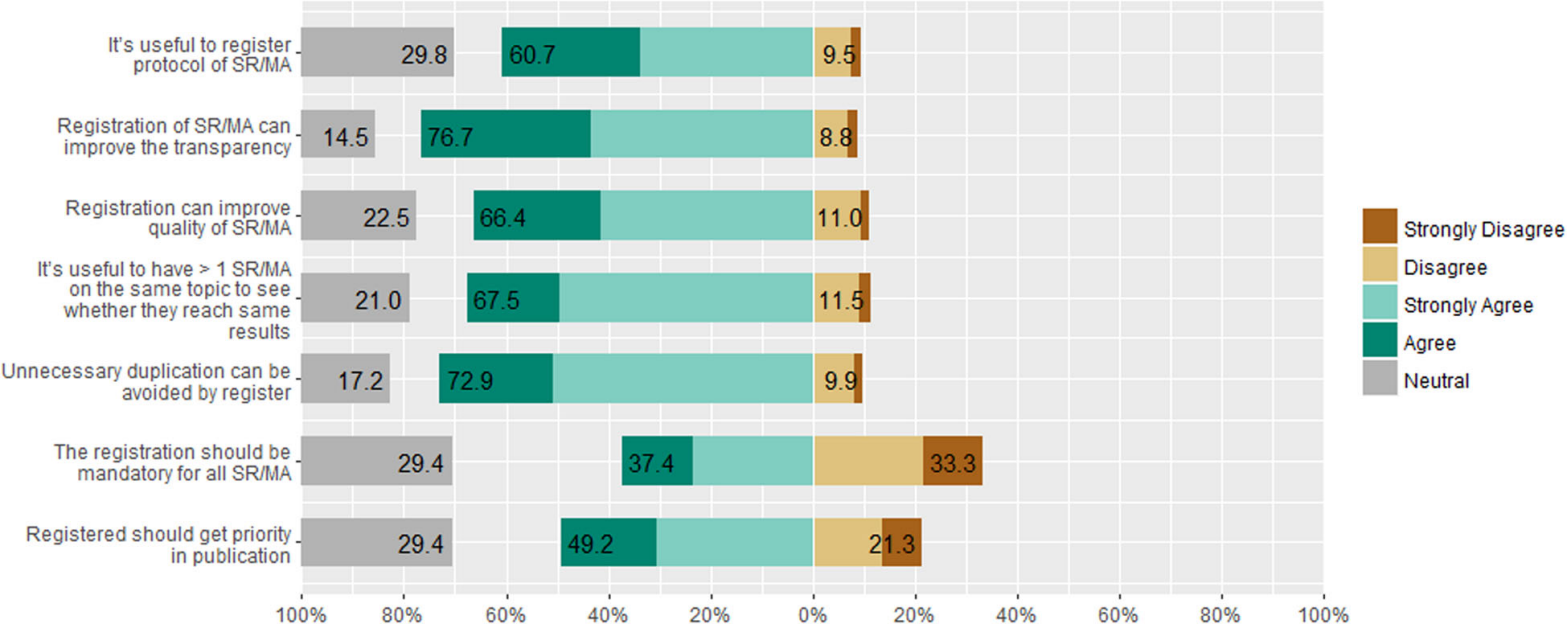
Authors’ opinions on registration protocol of systematic review/meta-analysis

A total of 76.7% of participants indicated that they believed that protocols improved the transparency, and 66.4% of them indicated that registration improved the quality of the SR/MAs. About 72.9% of the responders believed that protocol registration helped in avoiding unnecessary duplication of the reviews. However, 67.5% agreed that it was useful to have more than one SR/MA addressing the same research question to see whether the results would be consistent. Only 37.4% of participants agreed with making protocol registration mandatory for all SR/MAs and 49.2% of participants agreed that registered SR/MAs should have a priority in the publication process (Fig. 2).

### Authors’ opinions and suggestions to avoid duplications and idea theft

Among the responders, 37.4% believed that people were using the open-access databases that publish protocols to steal ideas of other researchers, 47.8% of them indicated that they have considered that the idea presented in their protocols could be stolen and 5.3% reported they personally experienced a situation where others stole ideas from their publicly registered protocol (Table 3).

**Table 3:** Stealing ideas

	*Number (%)*
Should the database (PROSPERO) hide all information and only publish when authors request before submission? Or they just publish the title to avoid duplication?, *n* = 234	
➣ Hide all information and publish when the authors request before submission	54 (23.1)
➣ Only publish the title to avoid duplication	129 (55.1)
➣ Other	51 (22)
Ever experienced a situation in which another group, who did not register their SR/MA protocol, publish before you have a paper based on a protocol identical to yours?, *n* = 246	16 (6.5)
If another group publishes a paper that is identical to your registered protocol before your team gets the publication and you find out their protocol registered after your protocol. What would be your next step?, *n* = 190	
➣ Identify the similarity and difference between your review and published ones and keep working on your own review	112 (59.3)
➣ Contact with both authors and editors as they possibly used your ideas	60 (31.2)
➣ Other	18 (9.5)
Ever heard of stealing ideas of registered protocol?, *n* = 256	55 (21.5)
Ever considered that the idea in your protocol could be stolen?, *n* = 253	121 (47.8)
Have others ever stolen your ideas from a protocol you registered?, *n* = 243	
➣ Yes	13 (5.3)
➣ No	130 (53.5)
➣ Donot know	100 (41.2)
Do you think that people are using the open register to steel other’s idea?, *n* = 230	
➣ Yes	86 (37.4)

Finally, we asked about the authors’ suggestions to avoid idea theft and protocol duplication. The majority of the responders suggested that PROSPERO should only publish the title of the protocol (55.1%), while some responders suggested that PROSPERO should hide all of the details about the registered protocol until the authors requested its publication prior to full manuscript submission (23.1%). The authors’ suggestions for avoiding idea theft and duplication are shown in detail in Supplementary Table 2.

## Discussion

Our results show insufficient knowledge among SR authors about the importance of protocol registration, which was directly reflected in their practice and opinions. About half of the participants believes that the main reason for not registering protocols, is that the other authors lack knowledge concerning obligation and importance to register the SR/MA protocols in advance. However, some authors have registered their protocol for some other reasons: It could be to preserve their rights of the idea or the journal they are targeting require them to register their protocol beforehand. Other reported reasons for not registering a SR protocol were: registration is not mandatory, carries no benefits, the process is time-consuming, and the fear of idea theft.

In this context, the myth that protocol registration istime-consuming or has no benefits has already been debunked [21]. Before conducting any SR/MA, researchers should scan the field for any ongoing or completed reviews on the same topic [22, 23]. However, many of the individual researchers or research groups do not register or publicly publish the protocols of their ongoing studies thus furtherly contribute to the problem of duplication. Accordingly, SR protocol registration has become an urgent need that has to be addressed by guideline developers and decision-makers [21].

Protocol registration is the way to alert different research groups that a related review is being conducted. This will not only help in preventing duplications, saving time and resources but also open the gate for collaborative work among the researchers with shared interests in a certain topic [21, 24]. Protocols development may take time. However, the step of writing the review’s protocol is critical in order to make sure that all investigators are on the same page so avoid wasting efforts or unintended bias [21, 24].

Additionally, registration will enhance confidence in the reported results by knowing that the methodology was determined in advance and making sure that it was not manipulated to suit the authors preference [21, 24]. Furthermore, a positive association between prospective registration and the methodological quality of SRs has been found [25]. The revised assessment of multiple SRs (R-AMSTAR) of registered reviews was higher than that for non-registered ones [25]. Similarly, the total preferred reporting items for SR/MAs (PRISMA) scores of registered reviews were significantly higher [25].

In 2013, PROSPERO has conducted an online survey to assess the experience of different users in registering their protocols [26]. Almost all (99%) respondents rated the PROSPERO navigation process as easy or very easy, and 79% of the participants have shown that they took 60 minuted or less to complete the registration form. These findings are inconsistent with the beliefs expressed by the respondents in our sample and eliminates the argument of the registration process being hard or taking too much time. The main reason for such belief could be a preconceived judgment without the actual understanding and knowledge of the process.

Regarding the fear of idea theft, nearly half of the participants suggested that it will be safer for them if the details of the ongoing SR/MA were blocked until the completion of their studies. In some datasets, this option is already available in order to assure authors as well as to limit the possibility of idea theft problem.

On the contrary, we have found that nearly 20% of authors have at least one paper that was registered but not published; either because they did not get results they anticipated or did not manage to finish the study. Furthermore, around 1% of our respondents never managed to publish any of their registered protocols. So, is it fair that someone keeps the idea forever for just thinking about it? [27].

A recent study by Tsujimoto et al. reported that around 26% of protocols registered in PROSPERO remained unpublished 5.4 years after registration [28]. Further, they reported that funding for SRs was a determining factor in the publication status of registered SRs [28, 29]. As some of the responders in our sample suggested, a possible solution for unpublished papers for registered protocols, is adding an option to allow for direct official contact with authors documented by the database itself. Authors of the ongoing SR/MAs may offer a collaboration or even confirm that they will not continue working on this idea. Another possible solution that was suggested by the responders and can be adopted by the databases registering SR protocols is to contact the authors in regular pre-defined intervals to make sure that they are still working on the registered study. If not, the authors can be given an option to either discarded the protocol from the database, to invite other potential authors to continue working on this topic, or to put up a notice that the authors are looking for collaborators that will help them complete the SR.

Some of our participants also suggested that collaboration between different databases registering protocols would be helpful in identifying duplicate ideas, as the same idea might be registered in two different databases by two different author teams. These potentially redundant protocols may pass screening unnoticed and get published, and the authors may discover too late that they have wasted their time and effort in an overlapping study.

The main limitation of this study was related to the low response rate 275/6650 (4.14%), raising the possibility of non-response bias. It is possible that many of the e-mails we sent ended up in the junk/spam folders, or that the e-mail addresses we used were functional but outdated and the authors do not use them anymore, as many e-mails were retrieved from manuscripts published 6–8 years ago. Our pilot testing indicated that the length of our questionnaire can be completed within a rational time, and we can therefore speculate that the length of the survey did not contribute to the low response rate.

Due to the limited sample size, our results are not necessarily generalizable [30, 31] in reverse to other some surveys who met good response rate [32, 33]. However, it has to be emphasized that surveys conducted via e-mail generally have lower response rates when compared to in-person surveys [34–37], which is a common problem in all online surveys in general [30, 31, 34–38]. Nonetheless, the conclusions we draw establish a strong foundation for further investigations related to SR protocol registration and provide ideas for fostering registration of such studies.

## Conclusions

About half of the surveyed systematic reviews’ authors have never registered any of their SRs’ protocols. About half of the participants believes that the main reason for not registering protocols, is that the other authors lack knowledge concerning obligation and importance to register the SR/MA protocols in advance. Therefore, rules and regulations should be made to direct protocol registration of any SRs beforehand.

Protocol registration of SRs can help minimize duplications and improve the overall quality of SRs. Fears about idea theft from open-access protocol registries make it mandatory to adopt more strict recommendations in this matter. In order to assure the authors that their ideas will be protected and their rights will be preserved, protocol registries should monitor such process and demand the authors of a potentially-duplicated protocol to give satisfactory reasons for registering such protocol. Moreover, collaborative efforts are needed to increase the recognition about the benefits of SR protocol registration and encourage authors to register their protocols.

## Supplementary information

**Additional file 1: Supplementary Table 1.** Checklist for Reporting Results of Internet E-Surveys (CHERRIES). **Supplementary Table 2.** Researchers suggestions about avoidance of duplication of SR/MA and stolen ideas (Open question and suggestions from each author). **Supplementary Fig. 1.** Nagasaki University ethics committee IRB waiver.

**Additional file 2.**

## Data Availability

The datasets used and/or analysed during the current study are available from the corresponding author on reasonable request.

## Abbreviations

SR/MAs: Systematic reviews and meta-analyses
CRD: Centre for Reviews and Dissemination
NIHR: National Institute for Health Research
DRMAs: “dose-response” meta-analyses
IF: Impact factor
