# Supplementary material for: Protocol registration issues of systematic review and meta-analysis studies: a survey of global researchers
Source: BMC Med Res Methodol. 2020 Aug 25;20:213. doi: 10.1186/s12874-020-01094-9 (PMC7448304; doi:10.1186/s12874-020-01094-9)

**Supplementary Table 1. Checklist for Reporting Results of Internet E-Surveys (CHERRIES)**

| *Item Category* | *Checklist Item* | *Explanation* |
| --- | --- | --- |
| Design | Describe survey design | Describe target population, sample frame. Is the sample a convenience sample? (In “open” surveys this is most likely.) |
| IRB (Institutional Review Board) approval and informed consent process | IRB approval | Mention whether the study has been approved by an IRB. |
|  | Informed consent | Describe the informed consent process. Where were the participants told the length of time of the survey, which data were stored and where and for how long, who the investigator was, and the purpose of the study? |
|  | Data protection | If any personal information was collected or stored, describe what mechanisms were used to protect unauthorized access. |
| Development and pre-testing | Development and testing | State how the survey was developed, including whether the usability and technical functionality of the electronic questionnaire had been tested before fielding the questionnaire. |
| Recruitment process and description of the sample having access to the questionnaire | Open survey versus closed survey | An “open survey” is a survey open for each visitor of a site, while a closed survey is only open to a sample which the investigator knows (password-protected survey). |
|  | Contact mode | Indicate whether or not the initial contact with the potential participants was made on the Internet. (Investigators may also send out questionnaires by mail and allow for Web-based data entry.) |
|  | Advertising the survey | How/where was the survey announced or advertised? Some examples are offline media (newspapers), or online (mailing lists – If yes, which ones?) or banner ads (Where were these banner ads posted and what did they look like?). It is important to know the wording of the announcement as it will heavily influence who chooses to participate. Ideally the survey announcement should be published as an appendix. |
| Survey administration | Web/E-mail | State the type of e-survey (eg, one posted on a Web site, or one sent out through e-mail). If it is an e-mail survey, were the responses entered manually into a database, or was there an automatic method for capturing responses? |
|  | Context | Describe the Web site (for mailing list/newsgroup) in which the survey was posted. What is the Web site about, who is visiting it, what are visitors normally looking for? Discuss to what degree the content of the Web site could pre-select the sample or influence the results. For example, a survey about vaccination on a anti-immunization Web site will have different results from a Web survey conducted on a government Web site |
|  | Mandatory/voluntary | Was it a mandatory survey to be filled in by every visitor who wanted to enter the Web site, or was it a voluntary survey? |
|  | Incentives | Were any incentives offered (eg, monetary, prizes, or non-monetary incentives such as an offer to provide the survey results)? |
|  | Time/Date | In what timeframe were the data collected? |
|  | Randomization of items or questionnaires | To prevent biases items can be randomized or alternated. |
|  | Adaptive questioning | Use adaptive questioning (certain items, or only conditionally displayed based on responses to other items) to reduce number and complexity of the questions. |
|  | Number of Items | What was the number of questionnaire items per page? The number of items is an important factor for the completion rate. |
|  | Number of screens (pages) | Over how many pages was the questionnaire distributed? The number of items is an important factor for the completion rate. |
|  | Completeness check | It is technically possible to do consistency or completeness checks before the questionnaire is submitted. Was this done, and if “yes”, how (usually JAVAScript)? An alternative is to check for completeness after the questionnaire has been submitted (and highlight mandatory items). If this has been done, it should be reported. All items should provide a non-response option such as “not applicable” or “rather not say”, and selection of one response option should be enforced. |
|  | Review step | State whether respondents were able to review and change their answers (eg, through a Back button or a Review step which displays a summary of the responses and asks the respondents if they are correct). |
| Response rates | Unique site visitor | If you provide view rates or participation rates, you need to define how you determined a unique visitor. There are different techniques available, based on IP addresses or cookies or both. |
|  | View rate (Ratio of unique survey visitors/unique site visitors) | Requires counting unique visitors to the first page of the survey, divided by the number of unique site visitors (not page views!). It is not unusual to have view rates of less than 0.1 % if the survey is voluntary. |
|  | Participation rate (Ratio of unique visitors who agreed to participate/unique first survey page visitors) | Count the unique number of people who filled in the first survey page (or agreed to participate, for example by checking a checkbox), divided by visitors who visit the first page of the survey (or the informed consents page, if present). This can also be called “recruitment” rate. |
|  | Completion rate (Ratio of users who finished the survey/users who agreed to participate) | The number of people submitting the last questionnaire page, divided by the number of people who agreed to participate (or submitted the first survey page). This is only relevant if there is a separate “informed consent” page or if the survey goes over several pages. This is a measure for attrition. Note that “completion” can involve leaving questionnaire items blank. This is not a measure for how completely questionnaires were filled in. (If you need a measure for this, use the word “completeness rate”.) |
| Preventing multiple entries from the same individual | Cookies used | Indicate whether cookies were used to assign a unique user identifier to each client computer. If so, mention the page on which the cookie was set and read, and how long the cookie was valid. Were duplicate entries avoided by preventing users access to the survey twice; or were duplicate database entries having the same user ID eliminated before analysis? In the latter case, which entries were kept for analysis (eg, the first entry or the most recent)? |
|  | IP check | Indicate whether the IP address of the client computer was used to identify potential duplicate entries from the same user. If so, mention the period of time for which no two entries from the same IP address were allowed (eg, 24 hours). Were duplicate entries avoided by preventing users with the same IP address access to the survey twice; or were duplicate database entries having the same IP address within a given period of time eliminated before analysis? If the latter, which entries were kept for analysis (eg, the first entry or the most recent)? |
|  | Log file analysis | Indicate whether other techniques to analyze the log file for identification of multiple entries were used. If so, please describe. |
|  | Registration | In “closed” (non-open) surveys, users need to login first and it is easier to prevent duplicate entries from the same user. Describe how this was done. For example, was the survey never displayed a second time once the user had filled it in, or was the username stored together with the survey results and later eliminated? If the latter, which entries were kept for analysis (eg, the first entry or the most recent)? |
| Analysis | Handling of incomplete questionnaires | Were only completed questionnaires analyzed? Were questionnaires which terminated early (where, for example, users did not go through all questionnaire pages) also analyzed? |
|  | Questionnaires submitted with an atypical timestamp | Some investigators may measure the time people needed to fill in a questionnaire and exclude questionnaires that were submitted too soon. Specify the timeframe that was used as a cut-off point, and describe how this point was determined. |
|  | Statistical correction | Indicate whether any methods such as weighting of items or propensity scores have been used to adjust for the non-representative sample; if so, please describe the methods. |

**Supplementary Table 2. Researchers suggestions about avoidance of duplication of SR/MA and stolen ideas (Open question and suggestions from each author)**

| Suggestions about avoidance of duplication of SR/MA |
| --- |
| - Mandatory registration of protocol. - Giving an ID for each title. These IDs should expired within 1 year if fail to publication as an article. - Highlight to journal editors and the scientific community that duplicates offer a more meaningful conclusion of the research question under investigation. - Having a website that only shows the published SR with a good-smart searching system. - Not revealing all details (just the title) and publishing full details of the protocol after publishing the results. - Journal authors asking for registration and whether another SR/MA was registered at the time the SR/MA submitted for publication was registered. - Alerted authors should be forced to contact the authors of first-registered protocol to verify: the actual need of an additional review on the same topic, or the potential collaboration among different groups. The response (even in case the collaboration is unfeasible) must be shared with Prospero (or other databases) and published for transparency. - Editors/peer reviewers should be experts in the field of research and know latest publications. Together with a statement of the authors on originality. - Try to include many different parameters for investigation that could make a manuscript unique. - Wider dissemination of the importance of registering. - Teach more people how to identify and develop a SR topic that is not redundant and potentially useful for end users. - Rigorous editorial review of SRs including checking if similar reviews have been started and when in databases of SRs. This needs to be enforced by the publishing journal. - Duplication is part of science, we cannot assume that all ideas are original. WE only should avoid plagiarism. - Registration of protocols is a good way to see whether others are doing the same review as you have planned. - Make the title and the key details (e.g. PICO) of the SR publicly available, but not the search strings, data extraction table, etc. - Examine PROSPERO and CAMRADES [basic science] as part of your scoping exercise. - PROSPERO should protect and identify post-registered similar protocols and request comprehensive discussion on how the second study is different and will add to the filed. - An integrated register system that prevents the registration of identical protocols. - Journals ensuring timely review by knowledgeable peers who can insist Authors address the "duplication issues. - An automated email sent by the database (e.g. PROSPERO) to the person that just registered a SR/MA with the top 10 similar registries it has. - Improve the control and responsibility of PROPERO. - The discussion section is often as interesting and important as the MA. - Some duplication is necessary e.g., when new evidence becomes available or when previous reviews were poorly done. |
| Suggestions about avoidance of stealing ideas of SR/MA |
| - Peer reviewers and publishers to show more awareness of this possibility. - Mandatory registration will solve the issue. So, another person cannot steal it. - I like the idea of only publishing titles, with the full protocol released only upon submission of the final review. - How about journals guarantee exclusive publication only for about 3-6 months to authors who registered protocol in specific topics. After permitted exclusive publication period, anyone can publish SR about these topics with duplicated protocol registration banned. - Prospective registration should be made mandatory for publication and same type of idea should not be accepted for registration. - Finish the review quickly! - Keeping the ideas confidential until after publication. - Work fast, act fast, stay silent. Do not let everyone know of what are you currently doing until your work is published. - Not revealing all details (just the title) and publishing full details of the protocol after publishing the results. - Partly publishing the protocol. When a manuscript is published the rest will be freely accessible. Possibly a request for info button on the protocol website. - Develop information tools to identify redundancy of protocols and, in case of overlap, authors should be alerted. - Editors should have access to registered protocols. In the submission process originality should be one of the items to state. - Engage end users (e.g., policy makers or guideline developers) of the SRs to ensure that no matter what, the SR will be useful for end users. I believe a high-quality SR will always be published even if there is another SR on the same topic or the idea "benign stolen". - Journal editors need to check the registries when accepting new SR manuscripts. Most changes in publication happen due to editor policies. If journal editor will not accept an unregistered SR, then authors will register it. - Enforce prior registration to make plagiarism transparent. - Journal editors should be more wary of SRs that are not registered and when manuscripts without registration are submitted these authors could be required to demonstrate they have checked registers to make sure that there are no similar registered titles. - Putting them in a registered protocol is itself a way of proving they were your ideas first. I guess more could be done to discourage (and/or punish) plagiarism. - Just make the title and short abstract available. - Transparency involves risks, but it is a necessary step. May the register (PROSPERO) could filter the protocols and detect situations where same (or very similar) titles and methods are submitted to them. - Do not use open registers. - Journals could check the protocol registry and the existence of another previous study with the same ideas before it accepts the article for publication. - Only publish those SR/MAs registered in PROSPERO or any other database. - Not registering full protocol. - This should be the responsibility of journal editors to check registration of the topic. And not publish something that is clearly duplicating other people's work. - Allow people to see the registered protocols only after a registration of the person himself. Therefore, if this person publishes something exactly the same as the protocol he was observing this could be tracked down. - Obtain a copy right agreement from the user before they download and view other's work, or just ensure to validate their credentials. - The academic community needs to be less internally competitive and more focused on producing high quality evidence for the benefit of patients. - PROSPERO should take responsibility to ensure similar SRs should not be registered if a pre-existing one is registered. Only registered SRs should be allowed to be published. |

Supplementary Table 2. SR/MA: systematic review meta-analysis.

***Supplementary Figure 1. Nagasaki University ethics committee IRB waiver***


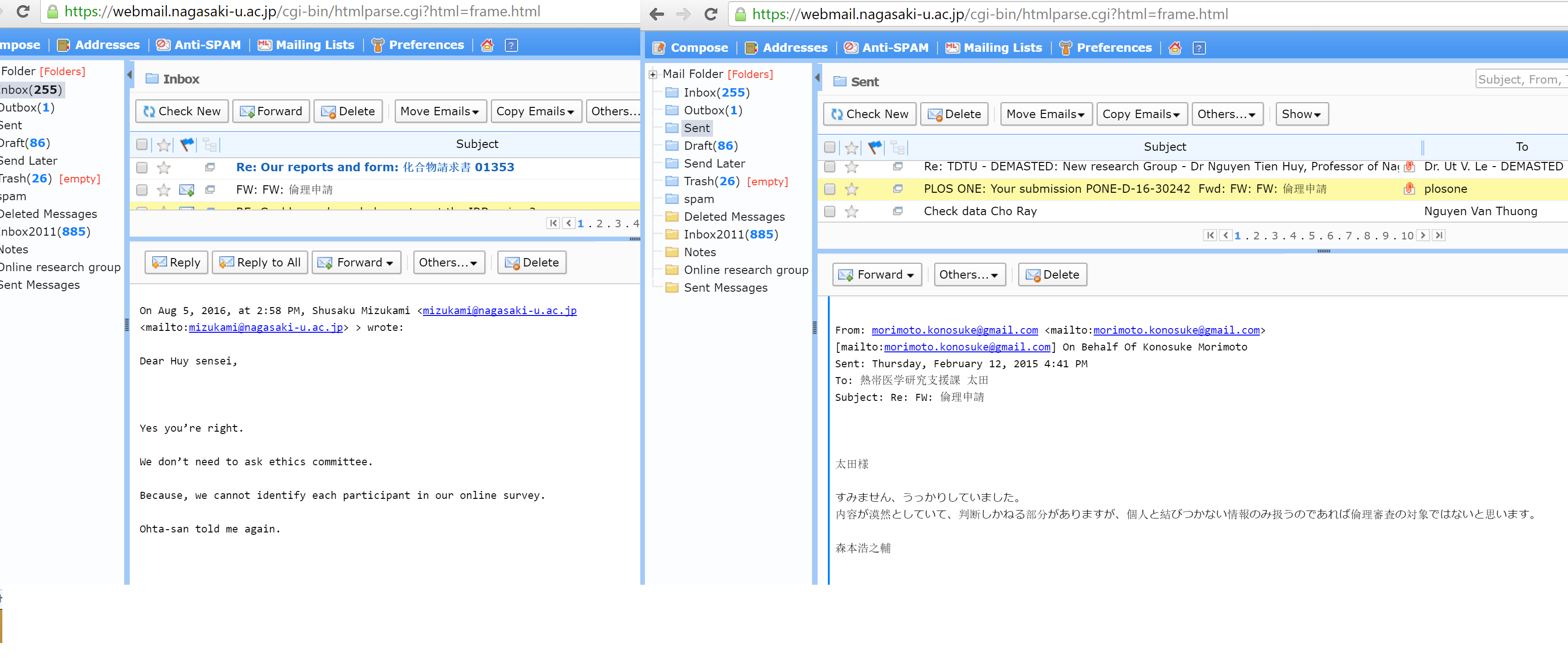

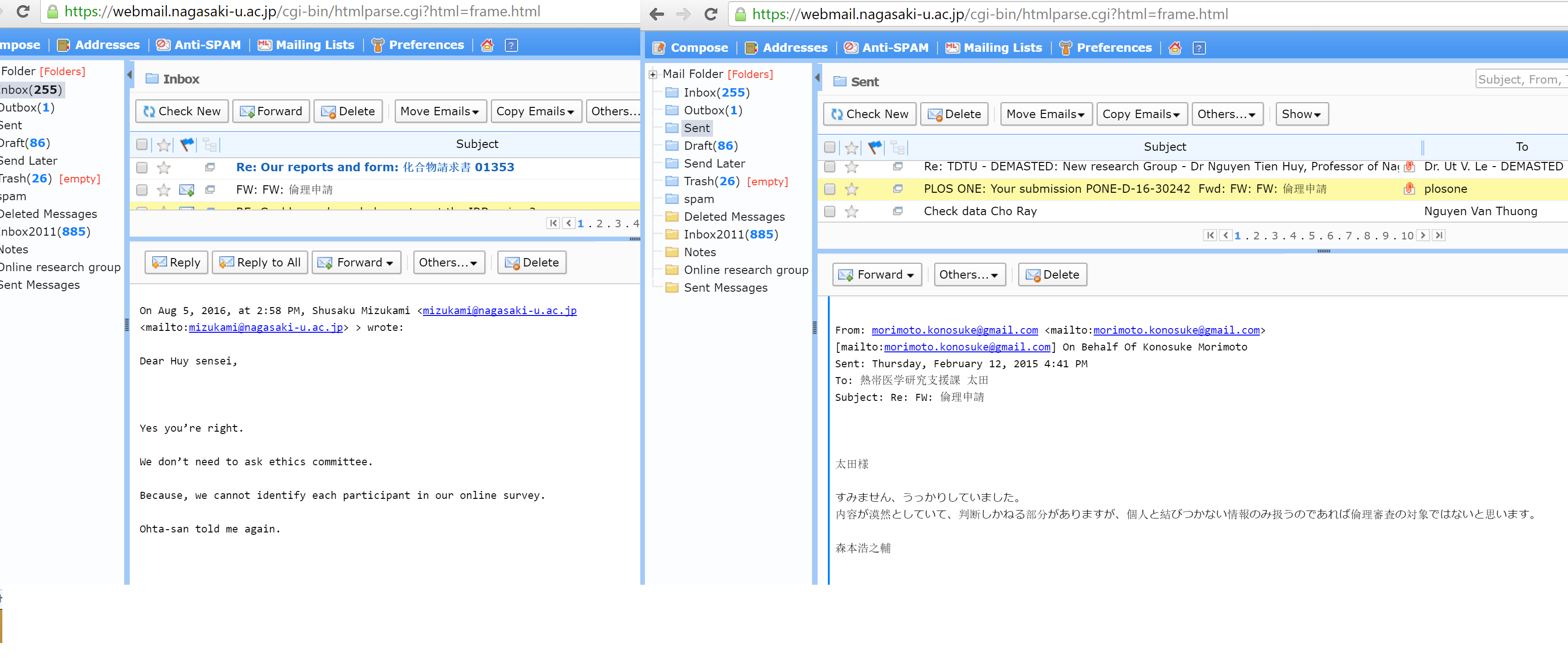


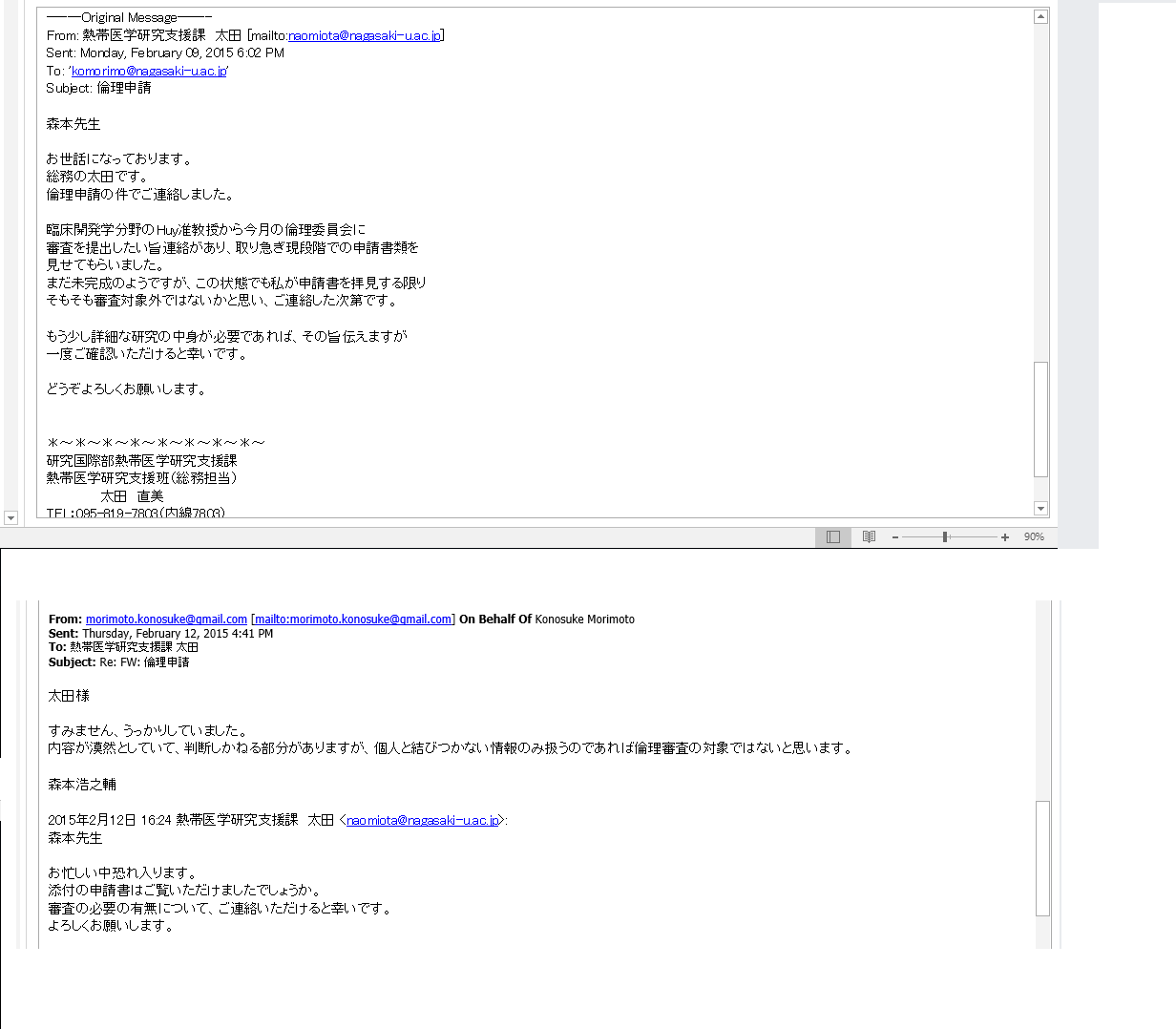

Supplement: Supplementary file 1 — Additional file 1: Supplementary Table 1. Checklist for Reporting Results of Internet E-Surveys (CHERRIES). Supplementary Table 2. Researchers suggestions about avoidance of duplication of SR/MA and stolen ideas (Open question and suggestions from each author). Supplementary Fig. 1. Nagasaki University ethics committee IRB waiver. [file 12874_2020_1094_MOESM1_ESM.docx]
