# Supplementary material for: Protocol registration issues of systematic review and meta-analysis studies: a survey of global researchers
Source: BMC Med Res Methodol. 2020 Aug 25;20:213. doi: 10.1186/s12874-020-01094-9 (PMC7448304; doi:10.1186/s12874-020-01094-9)
Supplement: Supplementary file 2 — Additional file 2. [file 12874_2020_1094_MOESM2_ESM.docx]

**Survey on Problems of Systematic review/Meta-analysis**

**By completing and submitting this survey, you are indicating your consent to participate in the study. Your participation is appreciated.**

**I. Demographic and professional information**

**Q0. Have you read information about the study given in the cover letter and agree to participate in this survey?**

**Q1. Year of birth**:

……………………

**Q2. Gender:**

□Male □Female

**Q3. How many years of experience do you have in Systematic Review/Meta-analysis (SR/MA)?** ......................

**Q4. In which country do you generally work on SR/MA?** ……………………

**Q5. What is your main research interest of SR/MA?**

□Basic science (such as *in vitro* study)

□Clinical trial

□Diagnostic accuracy

□Genetic association

□Epidemiology

□Other: please specify………….

**Q6. How many publications in SR/MA do you have?**

……………………

**Q7. What is the highest Impact Factor Journal of your publications in SR/MA?**

**□0-2 □2-5 □5-10 □10-20 □>20 □Do not know**

**Q8. What are your roles in published SR/MA?**

□Screening relevant studies

□Extracting data

□ Analyzing data

□Writing manuscript

□Correct and interpreting the result

□Supervising

□Other: please specify

**II. The registration of your SR/MA**

**Q9. Where have your previous SR/MA protocols been registered?** *(multiple choices)*

□PROSPERO University of York

□ Cochrane Database of Systematic Reviews (CDSR)

□Campbell Collaboration

□Other (please specific here): …………………………………

**Q10. How many percent of your SR/MA have been registered before starting?**

□100%

□80-100%

□50-80%

□20-50

□<20

□None of them

**Q11. How many percent of your SR/MA have been registered but never come to publish?**

□100%

□80-100%

□50-80%

□20-50

□<20

□None of them

**Q12. In your opinion, what could be the reason for not registering SR/MA? (check all that apply)**

□Submitting protocol takes too much time

□Afraid of others stealing my ideas

□Didn't know that it should be registered

□Have no idea about the benefit of registration the protocol

□It is not mandatory

□Others (please specify) -------------------------------------------------

**Q13. What can be the reason for not publishing registered SR/MA?**

**□**Have not reached the favorable conclusion

□Have not finished

□Pressure from sponsor/contractor

□Other (Please specify): ………………………………..

**Q14. Have you ever conducted SR/MA of basic biomedicine such as in vivo or in vitro studies?**

□Yes □No

**If yes, did you register the protocol?**

□ Yes □ No

**Q15. Have you been rejected by PROSPERO when you submitted a SR/MA protocol in basic biomedicine?**

□ Yes □ No

**If yes, what did you do when you got the rejection?**□ Contact PROSPERO University of York and explain

□ Revise and re-submit it to PROSPERO without any contact

□ Submit it to (please specific here): …………………………………….

□Other (please specific here): …………………………………

**Q16. How long does it take for the Prospero to accept your protocol?**

□ 1 to 3 working days

□ 4 to 6 working days

□ 1 week to 1 month

□ >1 month

**Q17. What was the average duration from registration to submission of your SR/MA manuscript?** Months…….

**Q18. Is the full registration of the full SR/MA protocol required in your institution?**

□Yes □No □Do not know

**Q19. Are your SR/MA protocols agreed by your sponsor?**

□Yes □No □I don’t inform the sponsor

**III. Your attitude toward registration protocol and problem on duplication**

**Q20. It is useful to register full protocol of SR/MA**

□ Strongly agree □Agree □Neutral □Disagree □Strongly disagree

**Q21. Registration of full SR/MA protocols can improve transparency**

□ Strongly agree □Agree □Neutral □Disagree □Strongly disagree

**Q22. Registration of full SR/MA protocols can improve their quality**

□ Strongly agree □Agree □Neutral □Disagree □Strongly disagree

**Q23. It is useful to have more than one SR/MA published on the same topic to see whether they reach the same results and conclusions**

□Strongly agree □Agree □Neutral □Disagree □Strongly disagree

**Q24. Unnecessary duplication can be avoided by using an open register**

□Strongly agree □Agree □Neutral □Disagree □Strongly disagree

**Q25. Should the database (Prospero) hide all information and only publish when the authors request before submission? Or they just publish the title to avoid duplication?**

□Hide all information and publish when the authors request before submission

□Only publish the title to avoid duplication

□Other (please specify)………………………………………………

**Q26. The registration of a protocol should be mandatory for all SR/MA before starting**

□Strongly agree □Agree □Neutral □Disagree □Strongly disagree

**Q27. Have you ever experienced a situation in which another group, who did not register their SR/MA, publish before you have a paper based on a protocol identical to yours?**

□Yes □No

**Q27.1 In this situation, what would you do next?**

A. Identify similarities and differences between your review and the published one, and complete your own review

B. Contact with both authors and editors as they possibly used your ideas

C. Other solution: ---------------------------------------------------------------------------------

**Q28. Have you ever experienced a situation in which another group, who register their SR/MA after you did, publish before you have a paper based on a protocol identical to yours?**

□Yes □No

**Q29: If another group publishes a paper that is identical to your registered protocol before your team get the publication and you find out their protocol registered after your protocol. What would be your next step?**

A. Identify the similarity and difference between your review and published ones and keep working on your own review

B. Contact with both authors and editors as they possibly used your ideas

C. Other solution: ---------------------------------------------------------------------------------

**Q30. A Registered protocol should get priority in publication**

□ Strongly agree □Agree □Neutral □Disagree □Strongly disagree

**Q31. Have you ever heard of stealing ideas from a registered protocol?**

□Yes □No

**Q32. Have you ever considered that the ideas in your protocol could be stolen?**

□Yes □No

**Q33. Have others ever stolen your ideas from a protocol you registered?**

□Yes □No □Do not know

If yes, how can you know that the idea was stolen? ................................

**Q34. Do you think that people are using registers to steal others peoples' ideas?**

□Yes □No

**Q35. Do you have any suggestions to avoid ideas being stolen? ..................**

**Q36. Do you have any suggestion regarding the avoidance of duplication of SR/MAs? ………………..**

**Thank you for your cooperation.**

**If you have any question, please don’t hesitate to contact us.**

Link of the online questionnaire:

**https://docs.google.com/forms/d/e/1FAIpQLSda7579tDckSNC44TmoZkkKAyLerYDsipFAYxpBvaP-3lAgww/viewform#responses**
